# TusDCB, a sulfur transferase complex involved in tRNA modification, contributes to UPEC pathogenicity

**DOI:** 10.1038/s41598-024-59614-2

**Published:** 2024-04-18

**Authors:** Yumika Sato, Ayako Takita, Kazutomo Suzue, Yusuke Hashimoto, Suguru Hiramoto, Masami Murakami, Haruyoshi Tomita, Hidetada Hirakawa

**Affiliations:** 1 Department of Bacteriology, Graduate School of Medicine, Gunma University, 3-39-22 Showa-machi, Maebashi, Gunma 371-8511 Japan; 2 Department of Infectious Diseases and Host Defense, Graduate School of Medicine, Gunma University, 3-39-22 Showa-machi, Maebashi, Gunma 371-8511 Japan; 3 Department of Clinical Laboratory Medicine, Graduate School of Medicine, Gunma University, 3-39-22 Showa-machi, Maebashi, Gunma 371-8511 Japan; 4 Laboratory of Bacterial Drug Resistance, Graduate School of Medicine, Gunma University, 3-39-22 Showa-machi Maebashi, Gunma, 371-8511 Japan

**Keywords:** Urinary tract infection (UTI), Bacterial pathogenesis, Virulence, tRNA modification, Biofilm, Drug resistance, Microbiology, Medical research

## Abstract

tRNA modifications play a crucial role in ensuring accurate codon recognition and optimizing translation levels. While the significance of these modifications in eukaryotic cells for maintaining cellular homeostasis and physiological functions is well-established, their physiological roles in bacterial cells, particularly in pathogenesis, remain relatively unexplored. The TusDCB protein complex, conserved in γ-proteobacteria like *Escherichia coli*, is involved in sulfur modification of specific tRNAs. This study focused on the role of TusDCB in the virulence of uropathogenic *E. coli* (UPEC), a bacterium causing urinary tract infections. The findings indicate that TusDCB is essential for optimal production of UPEC's virulence factors, including type 1 fimbriae and flagellum, impacting the bacterium's ability to aggregate in bladder epithelial cells. Deletion of *tusDCB* resulted in decreased virulence against urinary tract infection mice. Moreover, mutant TusDCB lacking sulfur transfer activity and *tusE*- and *mnmA* mutants revealed the indispensability of TusDCB's sulfur transfer activity for UPEC pathogenicity. The study extends its relevance to highly pathogenic, multidrug-resistant strains, where *tusDCB* deletion reduced virulence-associated bacterial aggregation. These insights not only deepen our understanding of the interplay between tRNA sulfur modification and bacterial pathogenesis but also highlight TusDCB as a potential therapeutic target against UPEC strains resistant to conventional antimicrobial agents.

## Introduction

Urinary tract infections (UTIs) are very common infectious disease, with an estimated 150 million UTI cases worldwide annually^1,2^. Uropathogenic *Escherichia coli* (UPEC) is known to be a major cause of UTIs, with more than 80% of UTI's attributed to this organism, and when UPEC enters the urinary tract and reaches the bladder, it causes cystitis^3,4^. In more severe cases, UPEC further ascends the urinary tract to the kidneys, causing pyelonephritis and sepsis^5^. Although various antimicrobial agents are used to treat UTIs caused by UPEC, UPEC infections are often refractory. It has been reported that 20% to 30% of patients with UTI will relapse within 6 months of antimicrobial therapy^6,7^. Therefore, there is concern that repeated treatment with antimicrobial agents may increase the risk of emergence of resistant strains. Since 2000, drug-resistant strains, including quinolone-resistant and ESBL (Extended spectrum β-lactamase)-producing bacteria, have been rapidly increasing and have become a major international problem^8,9^. For this reason, there is a need to create new therapeutic agents that are effective against various existing drug-resistant strains and new treatment strategies that can reduce the recurrence rate in the medical field.

UPEC secretes a variety of cytotoxic molecules such as hemolysin (Hly), cytotoxic necrotizing factor (CNF), and autotransporter toxin (SAT), and also internalizes urothelial cells, where they aggregate and establish biofilm-like polymicrobial structures, termed intracellular bacterial communities (IBCs)^10–13^. UPEC in IBCs are resistant to antimicrobial agents and innate immunity, and this mechanism of IBC formation is thought to be one of the reasons for the refractoriness of UPEC infections^14,15^. Fimbriae are known to be important factors in attachment to and invasion of host epithelial cells and in bacterial aggregation. Among them, type 1 and P fimbriae are the major fimbriae of UPEC, with type 1 fimbria being essential for infection of bladder epithelial cells, while P fimbria are proposed to contribute to infection of kidney epithelial cells^16,17^. The flagellum is also important for UPEC pathogenicity. The flagellum contributes to bacterial migration as well as fitness in the bladder and ascending from the bladder to the kidneys^18,19^. In addition, the flagellum has been shown to contribute to bacterial aggregation within both bladder and kidney epithelial cells^20,21^.

tRNAs are essential for the process of translation, in which amino acids are added based on codon information from mRNA. It is known that tRNAs synthesized in the cytoplasm are subsequently modified by various chemical molecules^22^. tRNA modifications are important for post-transcriptional regulation of genes, such as optimizing translation fidelity and efficiency^23^. Among these, sulfur modifications are well-studied and have been shown to play an important role in the regulation of various physiological functions, especially in mammalian cells. For example, defect of tRNA sulfur modification leads to mitochondrial diseases, while excessive sulfur modification is known to promote cancer development^24–26^. However, the role of tRNA sulfur modifications in the regulation of physiological functions in prokaryotes, including pathogenic bacteria, has been less studied than eukaryotic cells.

TusD, together with TusC and TusB, was identified as a protein constituting a sulfur transferase complex ^27^. The *tusD* gene is presumed to be transcribed as an operon together with its downstream genes *tusC* and *tusB* genes. One in vitro experiment using TusD, TusC, and TusB recombinant proteins showed that these proteins are involved in the sulfur modification to the uridine base at the anticodon wobble position (position 34) in tRNA^Lys^, tRNA^Glu^ and tRNA^Gln^^28^. This sulfur modification is proposed to be important for discrimination among Lys, Glu, and Gln codons in tRNA^Lys^, tRNA^Glu^ and tRNA^Gln^^29^. In *E. coli*, sulfur in cysteine residues is passed to IscS and then to TusA. TusDCB pulls sulfur from TusA and passes the sulfur molecule to the uridine base at position 34 via TusE and MnmA^28^. While TusDCB has been functionally analyzed in the above in vitro studies, the role of this protein within the infected host, including its virulence in *E. coli*, remains unclear.

We have been studying to identify the factors responsible for bacterial aggregation within urinary tract cells, leading to IBC formation, that contribute to the refractoriness of UPEC and to analyze their functions. In this study, we focused on TusDCB. The *tusDCB* defective strain showed similar growth performance to the parent strain in artificial urine medium, but had lower bacterial aggregation and UPEC virulence than the parent strain. Furthermore, the expression of type 1 fimbria and flagellum, which are important for infection of uroepithelial cells and bacterial aggregation, was decreased in the *tusDCB* defective strain, indicating that TusDCB is a factor supporting the expression of type 1 fimbria and flagellum and contributes to bacterial aggregation in uroepithelial cells and UTI.

## Results

### Deletion of the *tusDCB* gene reduces UPEC colonization in the bladder of mice

**Figure 1:**
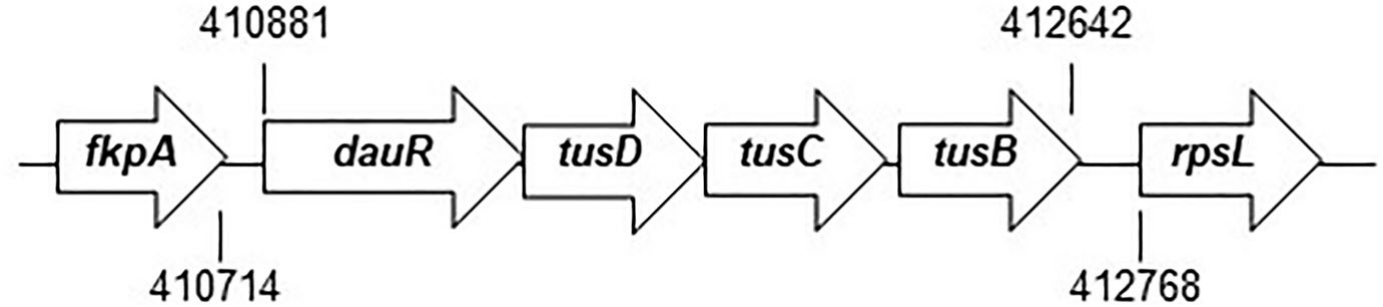
Locus of *dauRtusDtusCtusB* on the UPEC GU2018_CL13 chromosome. Arrows indicate transcription/translation direction. Numbers refer to nucleotide coordinates in the UPEC GU2018_CL13 genome (Accession number: (AP029000).

**Figure 2:**
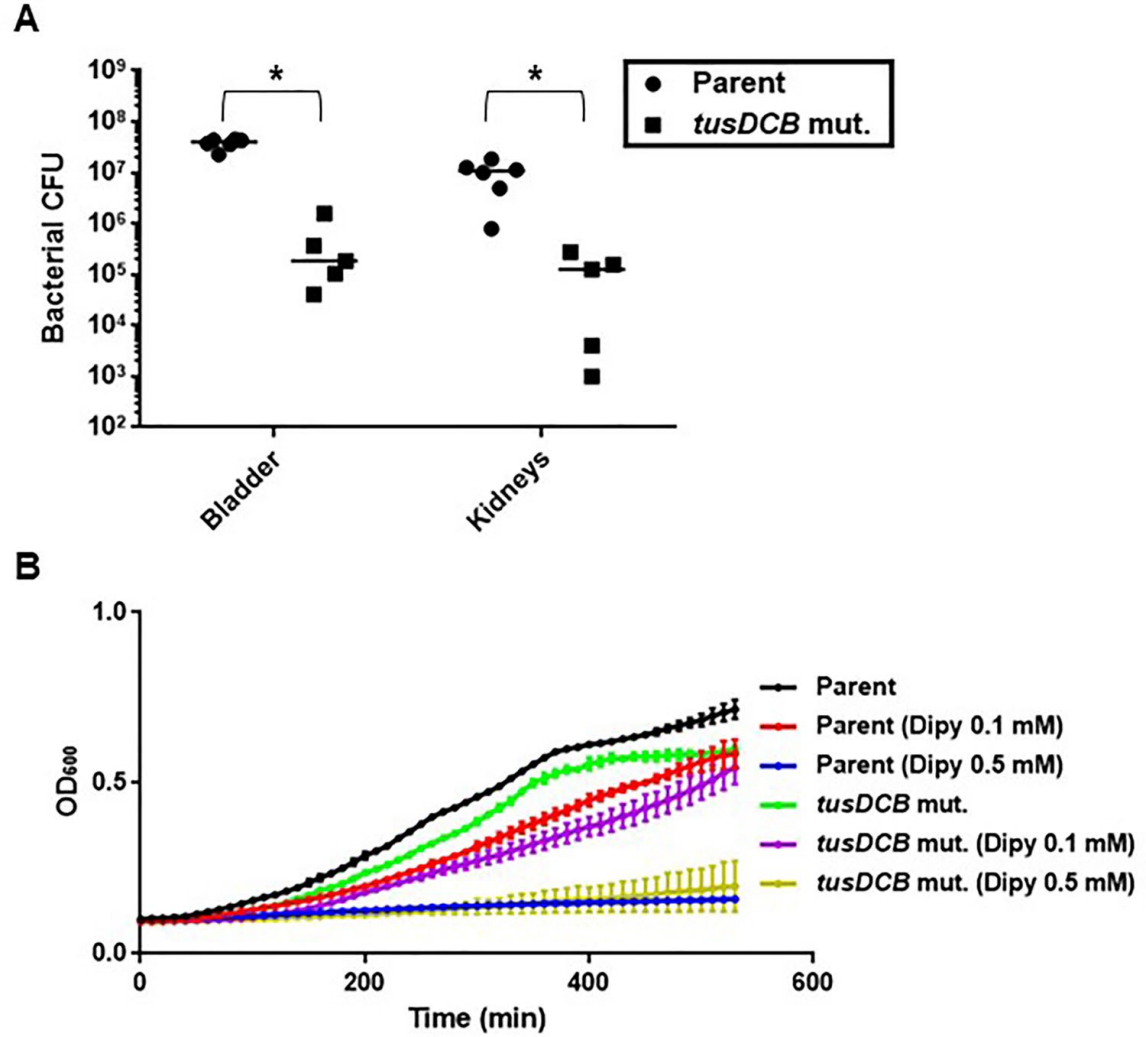
Colonization by the parent strain and *tusDCB* mutant in the bladders and kidneys of UTI mice and bacterial growth. (**A**) The female mice were infected with the parent strain (GU2018_CL13 parent) or *tusDCB* mutant (*tusDCB* mut.). At 48 h postinfection, cell numbers of bacteria isolated from the bladder and kidneys were determined as CFU. Each data point represents a sample from an individual mouse (n = 6 for the parent strain and n = 5 for the *tusDCB* mutant) (We guessed that the parent strain may be highly toxic then some of mice may die. Therefore, we used one more animal in the parent group than in the mutant group as a backup although no mice died in the parent strain infected group in this experiment). Horizontal bars show median values, *P < 0.05. Asterisks denote significance for values relative to the parent strain. The *P* value was determined by the Mann–Whitney test. (**B**) The parent strain and *tusDCB* mutant were cultured in mAUM containing 1 g/ L glucose with and without 2, 2'-dipyridyl (Dipy). Bacterial growth was monitored by measuring OD_600_. Data are means for two biological replicates; error bars indicate the ranges. We performed this assay twice, then similar results were obtained.

Initially, we evaluated the ability of the bacteria to colonize the bladder and kidneys of UTI mice using the *tusDCB* mutant and the UTI mouse model to determine the relationship between the TusDCB complex and the virulence of UPEC. The *tusC* gene (GU2018CL13_03790) is located immediately downstream of the *tusD* gene (GU2018CL13_03780), followed by the *tusB* gene (GU2018CL13_03800), which form an operon (Fig. 1). We constructed a *tusDCB* mutant that deletes the region from the *tusD* start codon to the *tusB* stop codon, as described in the Materials and methods section. The parent strain and the *tusDCB* mutant were transurethrally infected in female C3H/HeN mice, and the number of bacteria in the bladder and kidneys was determined at 48 h postinfection. The number of bacteria in the bladder and kidneys of mice infected with the *tusDCB* mutant was significantly lower than that of bacteria when infected with the parent strain (Fig. 2A). Since bacteria ascend the urinary tract to the kidneys after the bladder is infected, the decrease in the number of bacteria infecting the kidneys due to *tusDCB* deletion is assumed to be due to the decrease in the number of bacteria infecting the bladder.

We compared the growth of the parent strain and the *tusDCB* mutant. When the *tusDCB* mutant was cultured in mAUM (modified artificial urine medium)^30^, which mimics urine, no clear growth defect was observed, although the growth rate was slightly lower than that of the parent strain (Fig. 2B). Bacteria in the bladder are exposed to an iron-deficient environment because bladder epithelial cells produce lipocalins that trap the bacterial siderophores, then interfere with bacteria’s acquisition of iron (II)^31,32^. 2,2′-Dipyridyl is an iron (II) chelator, and the addition of this compound to the culture medium can deplete iron (II) in the medium. We compared the growth of the parent strain and the *tusDCB* mutant by adding 2, 2'-dipyridyl to mAUM. When a high concentration of 2, 2'-dipyridyl (0.5 mM) was added, growth was completely inhibited in both parent and *tusDCB* mutant strains. Growth of the parent strain was slightly inhibited when a low concentration (0.1 mM) of 2, 2'-dipyridyl was added, and the inhibitory effect of the *tusDCB* mutant was similar to that of the parent strain (Fig. 2B). Altogether, we concluded that the decrease in the number of infectious bacteria in the bladder caused by *tusDCB* deficiency is not solely due to a growth defect.

### The *tusDCB* gene is required for optimal aggregation of UPEC in bladder epithelial cells

**Figure 3:**
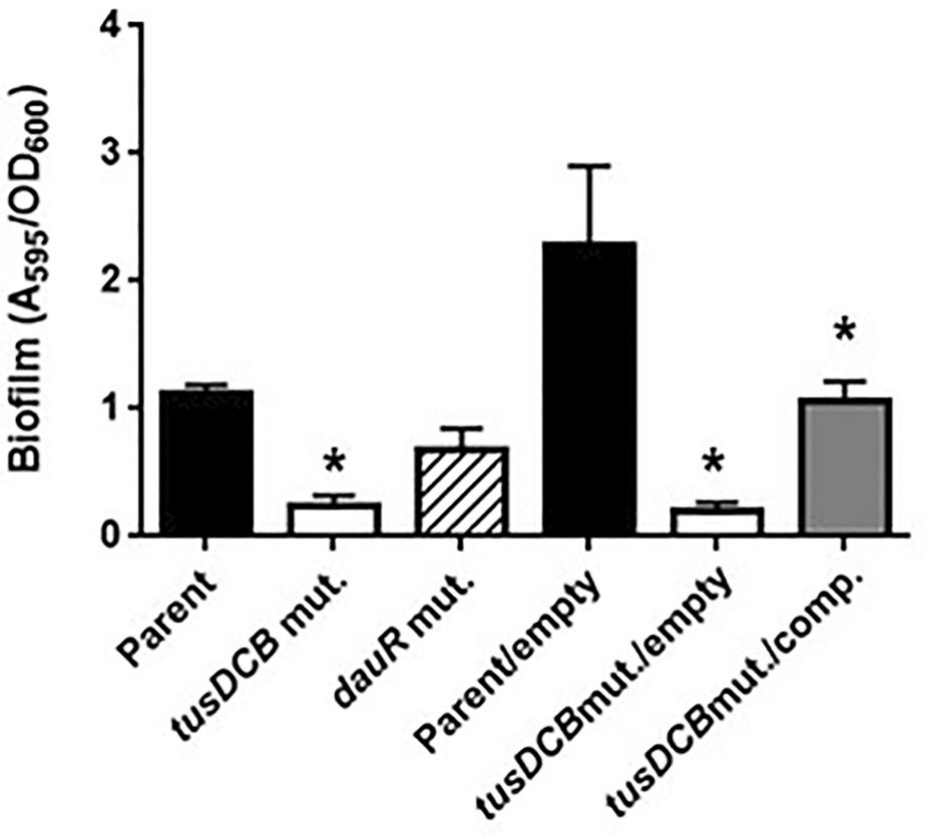
Biofilm formation on 96-well plates in the parent strain (GU2018_CL13 parent), the *tusDCB* (*tusDCB* mut.) and *dauR* mutants (*dauR* mut.), or the parent and the *tusDCB* mutant carrying the pTH18kr empty vector (Parent/empty and *tusDCB* mut./empty) or pTH18krdauRtusDCB, the *tusDCB* expression plasmid (*tusDCB* mut./comp.). Bacterial adhesion and aggregation were represented as A_595_ values normalized to OD_600_ of 1. Data plotted are the means of three biological replicates; error bars indicate standard deviations, *P < 0.05. Asterisks denote significance for values relative to parent or parent/empty. The *P* value was determined by the unpaired *t* test.

When UPEC infects the bladder, it invades bladder epithelial cells and aggregates^10^. From the result of experiments in UTI mice, we hypothesized that the *tusDCB* mutant may have a lower aggregation capacity than the parent strain. The ability of bacteria to aggregate is related to biofilm formation^33^. Therefore, we performed a biofilm assay using 96-well plates and crystal violet to estimate bacterial aggregation, and found that the amount of biofilm formed by the *tusDCB* mutant was significantly lower than the parent strain (Fig. 3). To confirm the contribution of the *tusDCB* gene in biofilm formation, the *tusDCB* gene was complemented by introducing the *tusDCB* expression plasmid pTH18kdaurRtusDCB into the t*usDCB* mutant. Since the *dauR* gene (GU2018CL13_03770), which encodes a transcriptional regulator, is located immediately upstream of the *tusD* gene in the same direction as *tusDCB*, and the start codon of *tusD* and a part of the stop codon of *dauR* overlap (Fig. 1), it is assumed that *tusDCB* and *dauR* form the same operon. Furthermore, promoter search revealed the promoter motif sequence upstream of the *dauR* start codon at positions 72–121. Based on the above, we speculated that gene expression of *tusDCB* depends on the promoter located upstream of *dauR*. Thus, to construct the *tusDCB* expression plasmid pTH18krdauRtusDCB, we cloned from 196 bases upstream of *dauR* including this promoter sequence to the region of stop codon of *tusB* into pTH18kr, a low-copy plasmid. When this plasmid was introduced into the *tusDCB* mutant, biofilm formation ability was significantly increased, although it did not recover to the level of the parent strain with empty vector (Parent/empty) (Fig. 3). To test whether the *dauR* gene is involved in biofilm formation, the *dauR* mutant was constructed and examined for the biofilm level. However, the mutant formed biofilms at the similar level to the parent strain, indicating that *dauR*, unlike *tusDCB*, is not involved in biofilm formation (Fig. 3).

**Figure 4:**
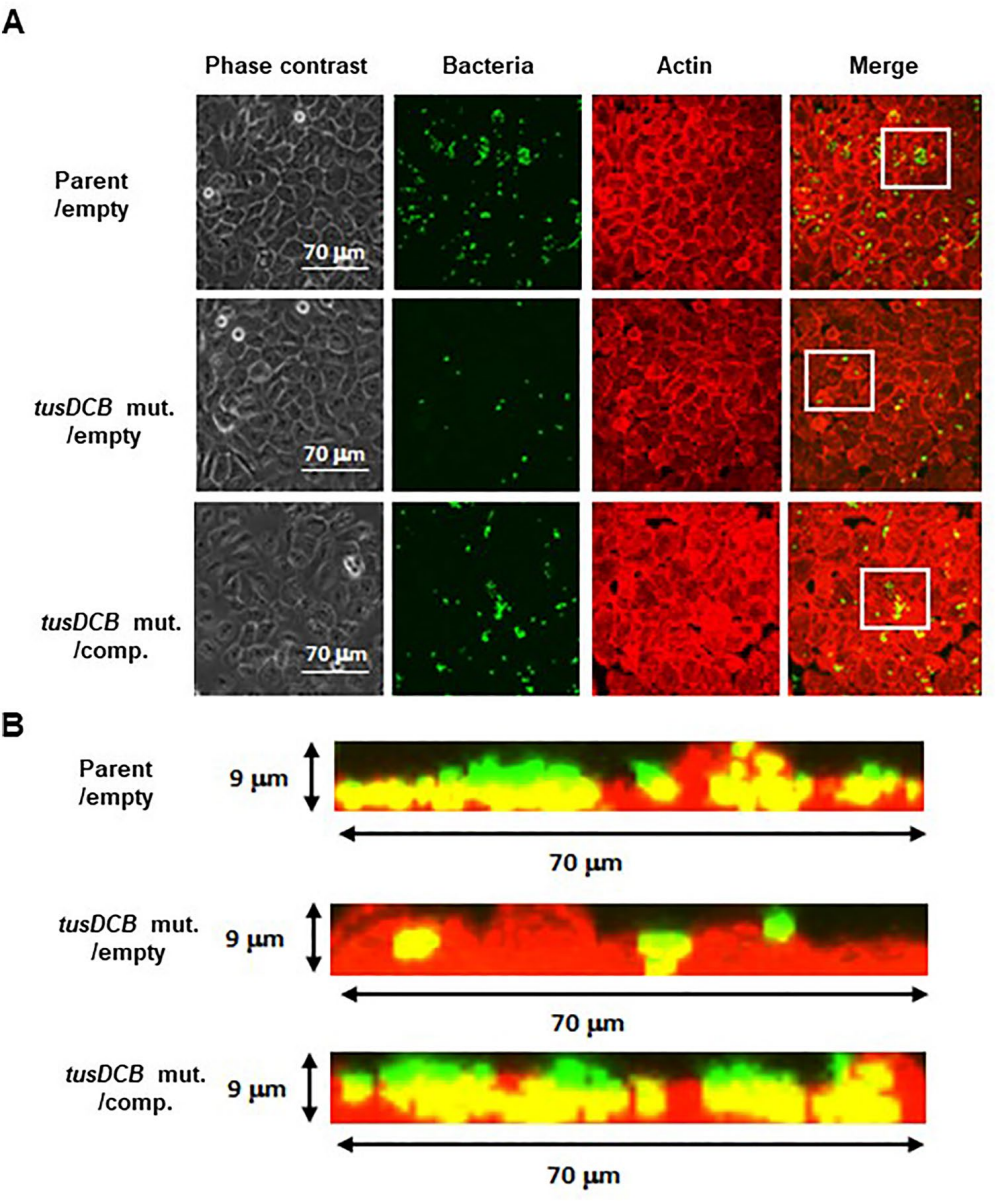
Aggregation within bladder epithelial cells (HTB-9) for the parent strain and the *tusDCB* mutant carrying the pTH18kr empty vector (Parent/empty and *tusDCB* mut./empty), and the *tusDCB* complementation strain (*tusDCB* mut./comp.). Bacteria carrying a green fluorescence protein (GFP) expression plasmid, pTurboGFP-B, and HTB-9 cells (Actin) stained with rhodamine-phalloidin were imaged with green and red fluorescence, respectively, using a 60 × objective. Images were taken from above (**A**), and cross-sectional images correspond to the white boxes (**B**). We performed this experiment twice, then similar results were obtained.

**Figure 5:**
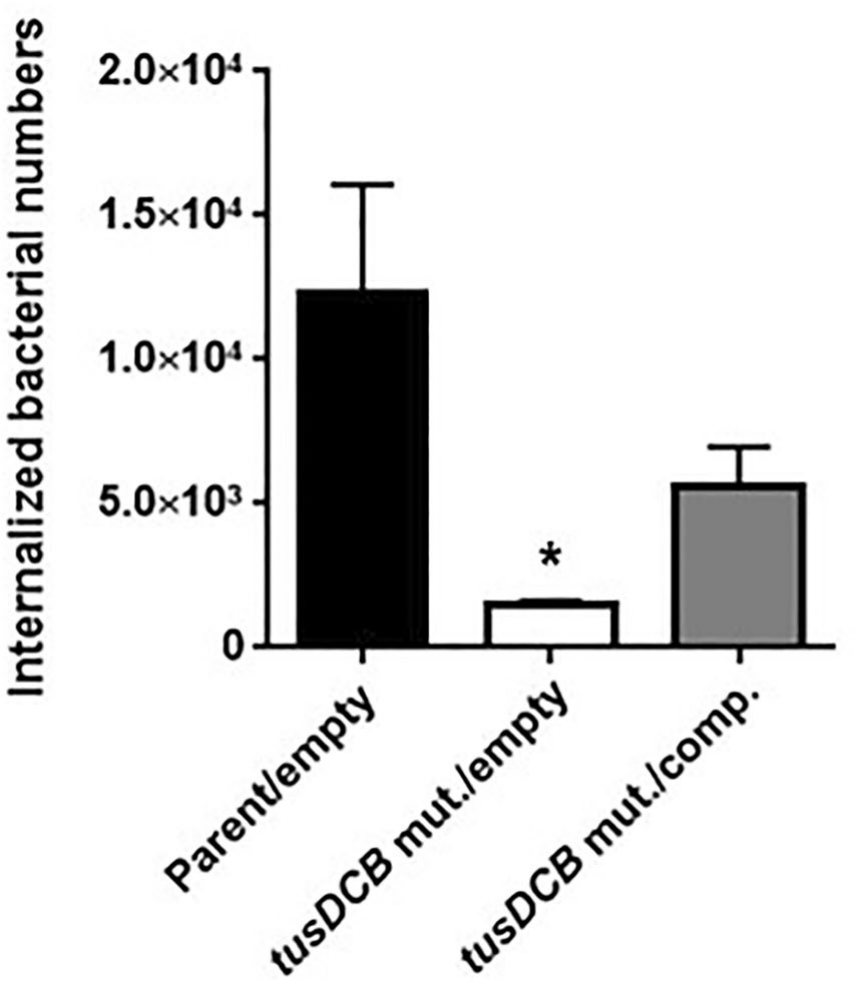
Internalization in balder epithelial cells (HTB-9) of the parent strain and the *tusDCB* mutant carrying the pTH18kr empty vector (Parent/empty and *tusDCB* mut./empty), and the *tusDCB* complementation strain (*tusDCB* mut./comp.). Numbers of internalized bacteria are represented. Data plotted are the means of three biological replicates; error bars indicate standard deviations, *P < 0.05. Asterisk denotes significance for values relative to parent/empty. The *P* value was determined by the unpaired *t* test.

We introduced the GFP expression plasmid pTurbo-GFP-B together with pTH18kr or pTH18krdauRtusDCB expression plasmids into the parent strain and the *tusDCB* mutant, and imaged bacterial cells when these strains were infected with bladder epithelial cells. In the parent strains, aggregated bacterial cells were observed in some places in the host cells. On the other hand, the *tusDCB* mutant carrying the empty vector exhibited fewer aggregates than the parent strain (Fig. 4A,B). We have confirmed that introduction of the pHT18krdauRtusDCB plasmid into the mutant restores the defective aggregation ability (Fig. 4A,B). We also compared the number of bacteria that entered the cells by gentamicin assay. The *tusDCB* mutant had a lower value than the parent strain (Fig. 5). The complementation of the *tusDCB* mutant with the pTH18krdauRtusDCB plasmid promoted bacterial internalization (Fig. 5). These results indicate that *tusDCB* contributes to UPEC aggregation in bladder epithelial cells.

### Deletion of *tusDCB* decreases type 1 fimbrial and flagellar expression

**Figure 6:**
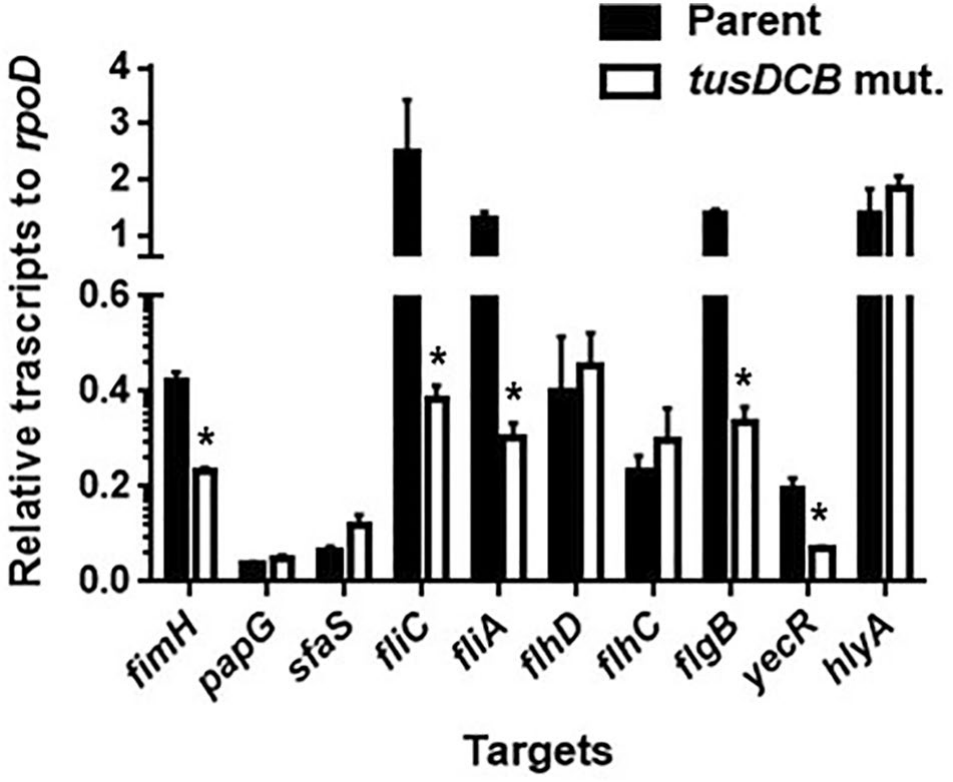
Transcript levels of fimbrial and flagellum-related genes and *hlyA* in the parent strain (GU2018_CL13 parent) and the *tusDCB* mutant (*tusDCB* mut.). Transcript levels were determined relative to that of *rpoD*. Data are means for four biological replicates; error bars indicate standard deviations, *P < 0.05. Asterisks denote significance for values relative to parent. The *P* value was determined by the unpaired *t* test.

Fimbriae are important for UPEC internalization and aggregation into bladder epithelial cells. Among them, type 1, P, and S fimbriae are the major fimbriae for UPEC, and groups of ORFs annotated as type 1, P, and S fimbriae, respectively^16,17,34^, are present on the chromosome GU2019-13. We selected *fimH* (GU2018CL13_39390), *papG* (GU2018CL13_40580) and *sfaS* (GU2018CL13_28390) from each group of genes encoding fimbriae and measured the transcript levels of these genes in parent and *tusDCB* mutant strains by quantitative PCR. The results showed that the transcript level of *fimH* in the *tusDCB* mutant was approximately half that of the parent strain, while the transcript levels of *papG* and *sfaS* were not significantly different between the parent and *tusDCB* mutant strains (Fig. 6).

Type 1 fimbria agglutinates guinea pig erythrocytes in the absence of mannose^35^. We compared the agglutination activity of guinea pig erythrocytes in a type 1 fimbria-dependent manner between the parent and the *tusDCB* mutant strains. In the absence of mannose, the aggregation titer of the parent strain was 256, whereas that of the *tusDCB* mutant was 128 (Table 1). Introduction of pTH18krdauRtusDCB into the *tusDCB* mutant increased the titer to the parental level (Table 1). On the other hand, no agglutination was observed in the presence of mannose in both the parent and mutant strains (Table 1), indicating that the agglutination in the absence of mannose is due to the activity of type 1 fimbria. These results suggest that *tusDCB* is involved in the type 1 fimbrial expression and activity.

**Table 1:** HA titers of the parent and the *tusDCB* mutant.

Strains	HA titers of guinea pig erythrocytes	
	-Mannose	+ Mannose
Parent (GU2018_CL13)	256	< 2
*tusDCB* mut. (GU2018_CL13ΔtusDCB)	128	< 2
Parent/empty	256	< 2
*tusDCB* mut./empty	128	< 2
*tusDCB* mut./comp	256	< 2

Parent/empty : GU2018_CL13 carrying pTH18kr.

*tusDCB* mut./empty : GU2018_CL13ΔtusDCB carrying pTH18kr.

*tusDCB* mut./comp. : GU2018_CL13ΔtusDCB carrying pTH18krdauRtusDCB.

**Figure 7:**
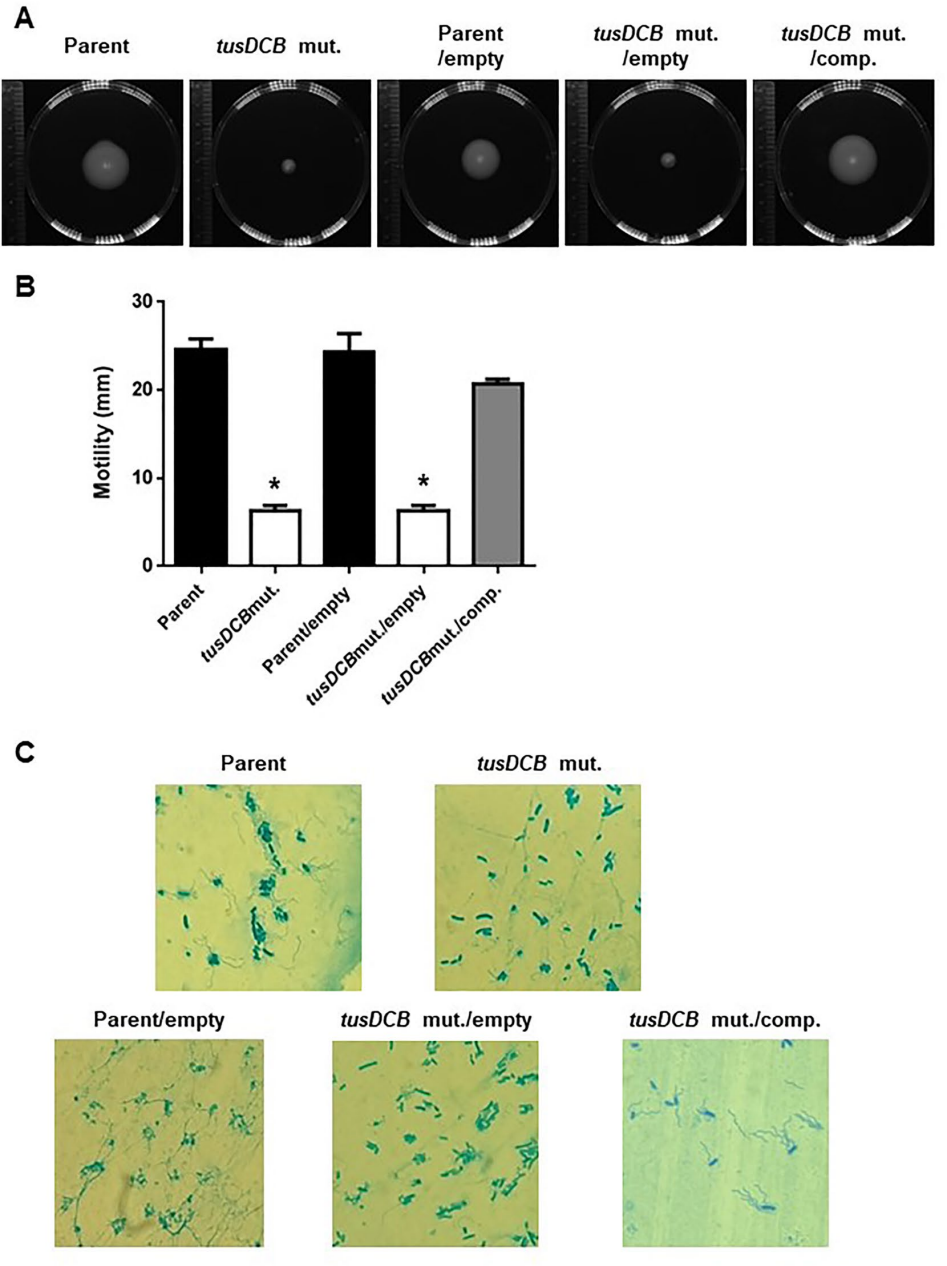
Motilities and flagellar production for the parent strain (GU2018_CL13 parent), the *tusDCB* mutant (*tusDCB* mut.), or the parent and the *tusDCB* mutant carrying pTH18kr (Parent/empty and *tusDCB* mut./empty) or pTH18krdauRtusDCB (*tusDCB* mut./comp.). (**A**) Bacterial migration on LB medium containing 0.25% agar. (**B**) Diameters reflecting bacterial migration on the agar. Data are means from three independent experiments; error bars indicate standard deviations, *, *P* < 0.05. Asterisks denote significance for values relative to parent. The *P* value was determined by the unpaired *t* test. (**C**) Flagella and bacterial cells were stained with Victoria blue/tannic acid were pictured using a 100 × objective. We performed this experiment twice, then similar results were obtained.

Flagellum is also important for UPEC aggregation in bladder epithelial cells^21^. We measured the transcript level of the *fliC* gene (GU2018CL13_19110), which encodes flagellin, the major protein of the flagellar structure^36^, and found that the *tusDCB* mutant showed approximately 6.5-fold lower level than the parent strain (Fig. 6). To compare the flagellar-dependent motility of the parent strain and the *tusDCB* mutant, the bacteria were inoculated on soft agar medium. A clear spread of the bacteria was observed with the parent strain, whereas no spread was observed with the *tusDCB* mutant, however bacterial spread was observed when pTH18krdauRtusDCB was introduced (Fig. 7A,B). Comparison of flagellar production between the parent and *tusDCB* mutant strains by flagellar staining showed that the mutant produced fewer flagella than the parent strain, and the ability to produce flagella was restored by introducing pTH18krdauRtusDCB into the *tusDCB* mutant (Fig. 7C).

Transcription of *fliC* is activated by the sigma factor FliA, which is activated by FlhDC, a master regulator complex of flagellar expression and *flhD* and *flhC* are co-transcribed as an operon^37^. Therefore, we measured the transcript levels of *fliA* (GU2018CL13_19120), *flhD* (GU2018CL13_19350), and *flhC* (GU2018CL13_19360) in addition to *fliC* by quantitative PCR analysis and found that, like *fliC*, the transcript level of *fliA* was approximately 4.3-fold lower in the *tusDCB* mutant than in the parent strain (Fig. 6). On the other hand, the level of *flhD* and *flhC* transcripts in the *tusDCB* mutant was comparable to that of the parent strain (Fig. 6). FlhDC also activates transcription of *flgB*, a gene encoding a flagellar component, and *yecR*, a gene not involved in flagellar production^38,39^. As with *fliA*, the transcript levels of these genes (*flgB*: GU2018CL13_03790/*yecR*: GU2018CL13 genome locus numbers 2093569–2,093,892) were lower in the *tusDCB* mutant than in the parent strain (Fig. 6). These results suggest that the ability of FlhDC to activate transcription of *fliA* leading to the *fliC* gene induction is reduced by *tusDCB* deletion.

We also measured the transcript level of the *hlyA* gene (GU2018CL13_40190) encoding hemolysin, however observed no significant differences between the parent and *tusDCB* mutant strains (Fig. 6).

### Sulfate transfer activity of TusD to TusE and MnmA is required for UPEC biofilm formation

**Figure 8:**
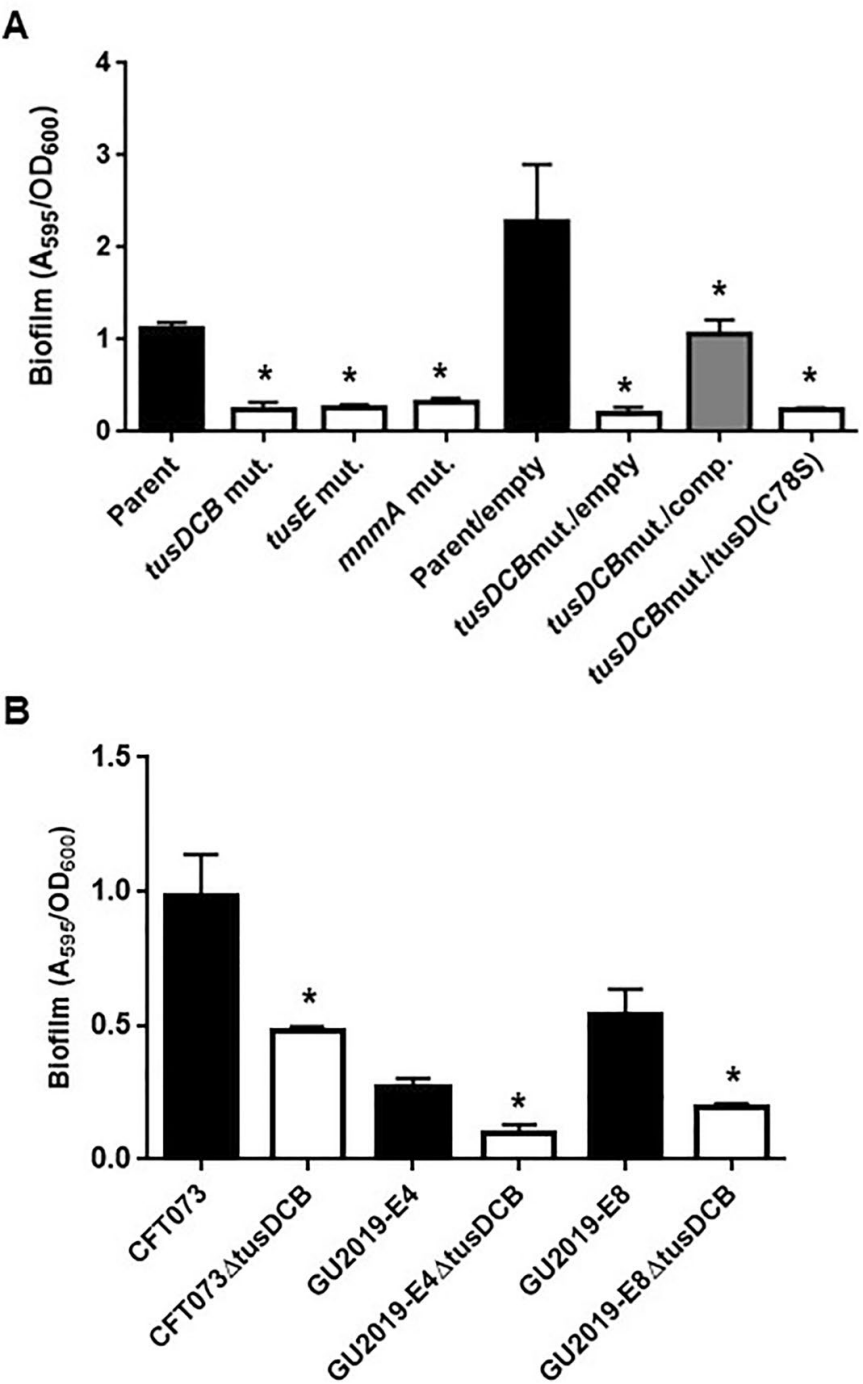
Biofilm formation on 96-well plates in the parent strain (GU2018_CL13 parent), the *tusDCB* (*tusDCB* mut.), *tusE* (*tusE* mut.) *mnmA* mutants (*mnmA* mut.), or the parent and the *tusDCB* mutant carrying pTH18kr (Parent/empty and *tusDCB* mut./empty), pTH18krdauRtusDCB (*tusDCB* mut./comp.) or pTH18krdauRtusD(C78S)tusCB (the *tusDCB* expression plasmid but the cysteine residue at the position 78 of TusD was replaced by the serine residue) (**A**) and the CFT073 strain, ESBL producers (GU2019-E4 and GU2019-E8) and their *tusDCB* mutants (**B**). Bacterial adhesion and aggregation were represented as A_595_ values normalized to OD_600_ of 1. Data plotted are the means of three biological replicates; error bars indicate standard deviations, *P < 0.05. Asterisks denote significance for values relative to parent or parent/empty. The *P* value was determined by the unpaired *t* test.

TusDCB passes a sulfur molecule to the uridine base at position 34 in the tRNA^Lys^, tRNA^Glu^ and tRNA^Gln^ via TusE and MnmA^28^. It is also known that Cys78 of TusD is an active site for sulfur transfer activity to pass the sulfur molecule to TusE. The TusD mutant (C78S) in which cysteine is replaced by serine has also been shown to lack sulfur transfer activity^27^. We performed biofilm assays using *tusE* and *mnmA* deletion mutants and the C78S mutant of TusD to verify whether the sulfur transfer activity of TusDCB is involved in biofilm formation. The *tusE* and *mnmA* mutants showed similar biofilm formation ability as the *tusDCB* mutant. The pTH18krdauRtusD(C78S)tusCB, a *dauR*-*tusDCB* complementation plasmid containing the C78S mutation in TusD, was introduced into the *tusDCB* mutant strain. However, biofilm formation ability was not restored, unlike when the pTH18krdauRtusDtusCB plasmid was introduced (Fig. 8A). These results suggest that the decrease in biofilm formation ability caused by *tusDCB* deletion is due to the loss of transfer activity of the sulfur molecule via TusE and MnmA.

### Deletion of *tusDCB* also decreases biofilm formation in the UPEC standard strain and highly pathogenic endemic multi-drug resistant strains

To test whether the role of TusDCB in pathogenicity and bacterial aggregation is conserved in other UPEC strains, CFT073 and UTI89, the commonly used UPEC standard strains in UPEC studies^40,41^, and ESBL-producing UPEC multidrug-resistant strains, GU2019-E4 and GU2019-E8 were selected. Strains GU2019-E4 and GU2019-E8 are ST131, a group of human highly pathogenic endemic strains^42^. The *tusDCB* gene sequences of these strains were compared and found to have more than 97% identity to the GU2018_CL13 strain (Supplementary Fig. S1). Among these strains, *tusDCB* gene was deleted in CFT073, GU2019-E4, and GU2019-E8 strains, and their biofilm forming ability was examined. As with strain GU2018_CL13, loss of *tusDCB* significantly reduced biofilm formation ability in CFT073, GU2019-E4 and GU2019-E8 strains (Fig. 8B). These results suggest that TusDCB are required for virulence and aggregation not only in GU2018_CL13 but also in other UPEC strains, including the highly pathogenic epidemic multidrug-resistant strains.

## Discussion

Sulfur modifications of tRNAs contribute to accurate codon recognition and optimization of translation efficiency^23^. The sulfur modification of the uridine molecule at position 34 of tRNA^Lys^, tRNA^Glu^ and tRNA^Gln^ is important for the identification of the third codon (A or G) of the amino acid^43,44^. The sulfur modification changes the uridine molecule at position 34 to a stabilized conformation called the 3'-endo form, which allows accurate recognition of the codons (A and G)^29,45^. The sulfur modification of the uridine molecule at position 34 has also been shown to bind more tightly to the A of the third codons of glutamine, glutamic acid, and lysine due to conformational constraints^46,47^. In other words, the CAA, GAA, and AAA codons are preferred for the addition of glutamine, glutamic acid, and lysine, respectively, in translation. In many organisms, both bacterial and eukaryotic, the codons for glutamine, glutamic acid, and lysine are dominated by CAA, GAA, and AAA compared to CAG, GAG, and AAG. In yeast and nematodes lacking this sulfur modification, significant translation stagnation at the glutamine and lysine codons was observed^48^. This sulfur modification has also been shown in mammalian cells, including humans, in relation to pathological conditions. For example, it is known that in humans, defect of sulfur modification of mitochondrial lysine tRNA results in a decrease in the total amount of mitochondrial proteins and the development of mitochondrial-derived mitochondrial disease (MERRE)^24,25^. In this study, we found that sulfur modification contributes to the virulence of UPEC.

The enzymes involved in sulfur modification differ between *E. coli* including UPEC and eukaryotes. In *E. coli*, TusDCB plays part of the role, and its homologues have been found in several γ-bacteria and several environmental bacterial species such as *Allochromatium vinosum*, *Chlorobaculum tepidum* (previously named *Chlorobium tepidum*) and *Thiobacillus denitrificans*^28,49^. However, it has not been confirmed in eukaryotic cells. In eukaryotic cells, Mtu1 is known, and although it shows about 37% homology with MnmA, a sulfur mediator downstream of bacterial TusDCB, it is distinctly different from MnmA^50,51^. Therefore, TusDCB is expected to be a promising potential target for the development of therapeutics to treat UPEC infections.

This study demonstrated that TusDCB is involved in the expression of type 1 fimbriae and flagella in UPEC, which contribute to infection of bladder epithelial cells and intracellular bacterial aggregation. The cap protein FimH of type 1 fimbriae binds to the uroplakin receptors of bladder epithelial cells, through its binding, allowing bacteria to adhere and efficiently enter the cell^15,16^. On the other hand, flagella contribute to the fitness and the formation of bacterial aggregation in the bladder^18,19,33,42^. Therefore, we speculate that the decrease in the number of bacteria infecting the bladder and the decrease in their aggregation in the epithelial cells caused by *tusDCB* defect involves at least the decreased expression of type 1 fimbriae and flagella.

The mechanism by which TusDCB contributes to the expression of type 1 fimbria and flagellum remains unknown. Transcript levels of *fimH*, which encodes the cap protein of type 1 fimbria, and *fliC*, which encodes flagellin, the major component of flagella, were measured by quantitative PCR, and the *tusDCB* mutant showed lower levels than the parent strain. However, since TusDCB is not a transcriptional regulator, it probably does not directly regulate the expression of the above genes.

Expression of *fliC* is induced by FlhDC, a master regulator of the flagellar gene expression, via the sigma factor FliA^37^. Our quantitative PCR analysis showed that *tusDCB* deletion reduced the transcript levels of *fliC* and *fliA*, but not *flhDC*. This suggests that TusDCB contributes to the gene expression of *fliA* in the hierarchical induction of gene expression in the FlhDC → FliA → FliC. Previous 3D structural analysis of FlhD and site directed mutagenesis experiments have identified some amino acid residues that are important for FlhD function^52^. Although none of the functional amino acid residues of FlhD contain glutamine, glutamic acid, or lysine, some of them are adjacent to the functional amino acid residues (e.g., Q27-D28-K29 and N61-Q62). Loss of TusDCB function may promote amino acid mutations in glutamine, glutamic acid, and lysine, which may alter the surrounding structure containing the functional amino acids and consequently diminish the function of FlhD. Alternatively, the delayed translation of glutamine, glutamic acid, and lysine adjacent to the functional amino acids might reduce the production of intact FlhD. The resulting reduced ability to induce expression of *fliA* decreases the transcript level of *fliC*. Although transcription of *flhDC* is regulated by several transcriptional regulators, including CytR by TosR^21,53^, no significant difference in transcript levels of *flhDC* was observed between the parent and *tusDCB* mutant strains, so the above transcriptional regulators are not affected by TusDCB or their effects are very low. Based on these data, we believe that TusDCB is at least required for normal translation of FlhD. It will be necessary to validate the amount of translation and frequency of amino acid mutations in FlhD.

Although less affected than flagellar production, measurement of *fimH* transcript levels and hemagglutination assay results indicated that defect on *tusDCB* also reduces the production of type 1 fimbriae. The gene cluster encoding the type 1 fimbriae structure, including *fimH,* consists of the *fim* operon, the transcription of which is controlled through phase variation by recombinases including FimB and FimE and transcriptional regulators such as Lrp and IHF^54^. TusDCB is assumed to contribute to the translation of any of these regulatory factors, and to the subsequent support of type 1 fimbrial expression.

In addition to flagellum and type 1 fimbriae, several other minor fimbria and adhesins contribute to UPEC pathogenicity, and it is not excluded that TusDCB may be involved in the production and activity of any of these factors. Currently, there does not appear to be any evidence that TusDCB has sulfur transfer targets other than tRNA^Lys^, tRNA^Glu^ and tRNA^Gln^. The TusDCB-mediated sulfur transfer reaction involves many factors including MnmA and TusA along with TusDCB, forming a sulfur transfer relay. Therefore, although the possibility that TusDCB has other targets cannot be completely excluded, it is speculated that the complexity of this reaction limits the sulfur transfer target to tRNAs^28^. The sulfur modification to tRNAs is initiated by the withdrawal of sulfur from the cysteine molecule by IscS. The pulled sulfur is also used in the biosynthesis of Fe-S clusters that affect the activity of various metabolic enzymes. The defect of TusDCB may indirectly affect Fe-S biosynthesis, the other sulfur flow destination, by terminating sulfur flow to tRNA^Lys^, tRNA^Glu^ and tRNA^Gln^. Another study showed that Tus proteins contribute to the expression of the global regulators RpoS and Fis^55^. Even though the target of TusDCB’s sulfur transfer activity is confined to tRNAs, it is involved in the regulation of diverse physiological functions. Therefore, a more comprehensive study is needed to understand the role of TusDCB in UPEC pathogenesis in more detail, including the identification of a comprehensive set of proteins involved in pathogenicity that are significantly affected by TusDCB.

The recent development of RNA analysis methods, including post-transcriptional modifications, has advanced this research field and increased the importance of this research area. This study not only sheds light on the intricate interplay between tRNA sulfur modification and bacterial pathogenesis but also unveils TusDCB as a potential target for therapeutic interventions. We believe that our findings will make a valuable contribution to the understanding of UPEC pathogenesis and have implications for the development of novel antimicrobial strategies.

## Materials and methods

### Bacterial strains, host cells and culture conditions

The bacterial strains and plasmids were listed in Table 2. The UPEC GU2018_CL13 strain was originally isolated from the urine and blood in a patient with pyelonephritis (Accession number: AP029000) (https://www.ncbi.nlm.nih.gov/nuccore/AP029000). The CFT073 strain is commonly used as an UPEC standard strain. This strain was originally isolated from the urine and blood of a woman with acute pyelonephritis in the United States^40^. GU2019-E4 is an ESBL-producing UPEC strain of the epidemic ST131 type previously reported^42^. We also used another UPEC ESBL producer of the epidemic ST131 type, designated GU2019-E8. This strain was isolated from the urine and blood in Japan, and is resistant to levofloxacin and gentamicin in addition to piperacillin, cefotaxime and aztreonam. Unless otherwise indicated, all bacteria were grown in LB (Luria–Bertani) medium. The cell growth was monitored by absorbance at 600 nm. For marker selection and maintenance of plasmids, antibiotics were added to growth media at the following concentrations; 30 µg/ml chloramphenicol, 50 µg/ml kanamycin and 150 µg/ml ampicillin. HTB-9 cells, the bladder epithelial cells were cultured in RPMI1640 medium containing 10% HyClone FetalClone III serum (HyClone Laboratories, Inc., Logan, UT, United States) at 37 °C and in an atmosphere of 5% CO_2_.

**Table 2:** Strains and plasmids used in this study.

Strain or plasmid	Relevant genotype/phenotype	Reference
Strains		
GU2018_CL13	Parent strain	This work
GU2018_CL13ΔtusDCB	*tusDCB* mutant from GU2018_CL13	This work
GU2018_CL13ΔdauR	*dauR* mutant from GU2018_CL13	This work
GU2018_CL13ΔtusE	*tusE* mutant from GU2018_CL13	This work
GU2018_CL13ΔmnmA	*mnmA* mutant from GU2018_CL13	This work
CFT073	ATCC700928	ATCC
CFT073ΔtusDCB	*tusDCB* mutant from CFT073	This work
GU2019-E4	Parent strain	^42^
GU2019-E4ΔtusDCB	*tusDCB* mutant from GU2019-E4	This work
GU2019-E8	Parent strain	This work
GU2019-E8ΔtusDCB	*tusDCB* mutant from GU2019-E8	This work
Plasmids		
pKO3	Temperature sensitive vector for gene targeting, *sacB*, Cm^R^	^56^
pTH18kr	Low copy plasmid; Km^R^	^57^
pTH18krdauRtusDCB	*tusDCB* expression plasmid; Km^R^	This work
pTH18krdauRtusD(C78S)tusCB	*tusDCB* (TusD C78S) expression plasmid; Km^R^	This work
pTurboGFP-B	GFP expression plasmid; Ap^R^	Evrogen

Cm^R^ : Chloramphenicol resistance, Km^R^ : Kanamycin resistance, Ap^R^ : Ampicillin resistance.

### Cloning and mutant constructions

An in-frame deletion mutant of *tusDCB* was constructed by sequence overlap extension PCR according to a strategy described previously^56^, with primer pairs, delta1 / delta2 and delta3 / delta4 primers for each gene as described in Table 3. The upstream flanking DNA included 450 bp and the first two amino acid codons for *tusD*. The downstream flanking DNA included the last two amino acid codons for *tusB,* the stop codon, and 450 bp of DNA. This deletion construct was ligated into BamHI and SalI-digested temperature sensitive vector pKO3^56^ and introduced into the UPEC strains. Then, sucrose-resistant/chloramphenicol-sensitive colonies were selected at 30 °C. We also constructed *dauR*, *tusE* and *mnmA* mutants using primer pairs dauR-delta1/dauR-delta2/dauR-delta3/dauR-delta4, tusE-delta1/tusE-delta2/tusE-delta3/tusE-delta4 and mnmA-delta1/ mnmA-delta2/mnmA-delta3/ mnmA-delta4, respectively.

**Table 3:** Primers used in this study.

Primer	DNA sequence (5’ – 3’)	Use
tusDCB-delta1	gcgggatccggagcggatagcagcgtttc	*tusDCB* mutant construction
tusDCB -delta2	caacgatcccgccatcaccaggcacgcattacttatcttgccc	*tusDCB* mutant construction
tusDCB -delta3	caggggcaagataagtaatgcgtgcctggtgatggcgggatcg	*tusDCB* mutant construction
tusDCB -delta4	gcggtcgactctttaacgccggagcagtc	*tusDCB* mutant construction
dauR-delta1	gcggcggccgctcgacggtaaagagttcgac	*dauR* mutant construction
dauR-delta2	aacgcattacttatcttgcccctgggttaaaagcgacctggac	*dauR* mutant construction
dauR-delta3	ttcatgtccaggtcgcttttaacccaggggcaagataagtaatg	*dauR* mutant construction
dauR-delta4	gcggtcgactaatccctcgcggccagcc	*dauR* mutant construction
tusE-delta1	gcgggatccggaatgggtgacgtaatcgc	*tusE* mutant construction
tusE -delta2	acggattttcgtatccgttaaatctctttaccttcgaagatcag	*tusE* mutant construction
tusE -delta3	tgctgatcttcgaaggtaaagagatttaacggatacgaaaatcc	*tusE* mutant construction
tusE -delta4	gcggtcgacaacggccccggaaaagacc	*tusE* mutant construction
mnmA-delta1	gcgggatccgttgaagagacgattaatgg	*mnmA* mutant construction
mnmA -delta2	aaagataataatcagaccggcagggtttcagacattggatcac	*mnmA* mutant construction
mnmA -delta3	tgagtgatccaatgtctgaaaccctgccggtctgattattatc	*mnmA* mutant construction
mnmA -delta4	gcggtcgaccatagatagcagccatcgc	*mnmA* mutant construction
dauR-F	gcgggatccgcgaaagccgcagactctgc	pTH18krdauRtusDCB construction
pTHtusB-R	gcgaagctttcaccaggccatctggctgg	pTH18krdauRtusDCB construction
tusD-C78S-F	gtggcgctgaatatctccgtagcggcggcattac	C78S mutant construction
tusD-C78S-R	gtaatgccgccgctacggagatattcagcgccac	C78S mutant construction

To construct the *tusDCB* complementation plasmid pTH18krdauRtusDCB, we PCR-amplified the region from 196 bases upstream of *dauR* to the stop codon of *tusB* with dauR-F and pTHtusB-R primers, and ligated into the BamHI and HindIII sites in pTH18kr, the low-copy-number plasmid^57^. The *dauR* gene is located upstream of the *tusD* gene and forms an operon with *tusDCB*. Therefore, we presumed that the *tusDCB* is transcribed under the promoter control of the *dauR* gene. For this reason, we included *dauR* and its upstream region to express *tusDCB* on a plasmid.

We also constructed the C78STusD expression plasmid pTH18krdauRtusD(C78S)tusCB by site-directed mutagenesis of pTH18krdauRtusDCB. The mutation was generated by using the primers tusD-C78S-F and tusD-C78S-R and.

KOD FX Neo polymerase (Toyobo Co., Ltd., Osaka, Japan) as previously described^58^. All constructs were confirmed by DNA sequencing.

### Urinary tract infections in mice

We estimated UPEC virulence using a UTI mouse model as previously described^33^. Bacterial suspensions in phosphate-buffered saline (PBS) (1 × 10^8^ CFU) were administered to 8-week-old C3H/HeN female mice via transurethral catheterization. The numbers of CFU in the bladder and kidneys 48 h postinfection were determined by counting colonies grown on XM-G agar. All animal studies were approved by the Animal Research Committee of Gunma University (approval number 19-094).

### Static biofilm assay

Levels of biofilm formation on 96-well plates were quantified as described previously with slight modifications^59^. Bacteria were cultured at 37 °C for 24 h in LB medium. Each culture was diluted into Dulbecco’s Modified Eagle Medium at a 1:100 ratio, and 2.4 × 10^4^ cells/well were seeded into the 96-well flat bottom polystyrene plate. The plate was then incubated at 37 °C and in an atmosphere of 5% CO_2_ for 24 h. Bacterial cells attached to the plate were stained with crystal violet and absorbance at 595 nm (A_595_) was measured. Bacterial aggregation ability was quantified as the A_595_ normalized to an OD_600_ of 1.

### Imaging of bacteria invading bladder epithelial cells and quantification of internalized bacteria

The bacteria in HTB-9 cells were imaged using confocal microscopy, as previously described^33^. A UPEC strain carrying a green fluorescence protein (GFP) expression plasmid, pTurboGFP-B (Evrogen, Moscow, Russia), was inoculated into cultured HTB-9 cells. The HTB-9 cells were stained with rhodamine-phalloidin (Life Technologies, Carlsbad, CA, USA). Fluorescent images were acquired on an Olympus FV10i-DOC microscope and processed using FV10-ASW software (Olympus Corp., Tokyo, Japan).

The number of bacteria to invade bladder epithelial cells (HTB-9) was determined by gentamicin protection assay. HTB-9 cells were cultured to confluence in 24-well plates and then we inoculated ~ 5.0 × 10^6^ bacteria into ~ 5.0 × 10^5^ host cells. After incubation for 2 h, the wells were washed once with PBS^+^ (PBS containing 0.5 mM Mg^2+^ and 1 mM Ca^2+^) and incubated them in the presence of gentamicin at 100 µg/ml for another 2 h. The wells were washed twice with PBS^+^, and the cells were lysed by 0.1% Triton X-100 and plated to determine bacterial numbers.

### Hemagglutination assays

To estimate the activity of type 1 fimbria, we tested the hemagglutination titers of guinea pig as previously described^33^.

### RNA extraction and quantitative real-time PCR analyses

Bacteria were grown to the late-logarithmic growth phase (optical density at 600 nm [OD_600_] ~ 0.7) in LB medium. Total RNA extraction and cDNA synthesis were performed by using the Monarch Total RNA Miniprep kit (New England Biolabs, Ipswich, MA) and ReverTra Ace qPCR RT Master Mix with gDNA Remover (Toyobo Co. Ltd., Osaka, Japan). Real-time PCR mixtures included 2 ng of cDNA and 160 nM primers in Thunderbird Next SYBR qPCR Mix (Toyobo). Constitutively expressed *rrsA* and *rpoD* genes were used as an internal control. The primers are listed in Table 4.

**Table 4:** Primers used for quantitative PCR in this study.

Primer	DNA sequence (5’ – 3’)	Primer	DNA sequence (5’ – 3’)
rrsA-qPCR-F	cggtggagcatgtggtttaa	rrsA-qPCR-R	gaaaacttccgtggatgtcaaga
rpoD-qPCR-F	caagccgtggtcggaaaa	rpoD-qPCR-R	gggcgcgatgcacttct
fimH-qPCR-F	tgcccgcaggtttgattc	fimH-qPCR-R	ccatggcacaaagcccata
papG-qPCR-F	aagccgaccctggacctt	papG-qPCR-R	acggtttgaaccacattttgc
sfaS-qPCR-F	cacaatttccggcgctaaa	sfaS-qPCR-R	gccagtagagcggcaaaaag
flhD-qPCR-F	gacaacgttagcggcactga	flhD-qPCR-R	ttgattggtttctgccagctt
flhC-qPCR-F	tcaggaagcgcgggatatt	flhC-qPCR-R	gagcgcccagggtgatc
fliA-qPCR-F	cgagcgtggaacttgacgat	fliA-qPCR-R	cgacggcattaagtaacccaat
fliC-qPCR-F	tccatcgacaaattccgttct	fliC-qPCR-R	gcggaatccagacggttct
flgB-qPCR-F	tcaggctcgcgatatcgatt	flgB-qPCR-R	ccgtccacgttgcatgact
yecR-qPCR-F	ggcccatgtgagcgaagt	yecR-qPCR-R	cctgatcataaaccaaccgaaca
hlyA-qPCR-F	ggcacggcgattactaaacag	hlyA-qPCR-R	cgttcggtgaggccaatg

### Motility assay

Bacteria were statically grown overnight at 37 °C. The bacterial cultures (2 μl) were spotted onto LB medium containing 0.25% agar and incubated for 16 h at 30 °C.

### Flagellar stain

Bacteria were cultured for 24 h at 30 °C in Heart Infusion medium containing 1.5% agar. Flagella were stained with Victoria blue/tannic acid solution as previously described^20^.

### Supplementary Information

Supplementary Information.

## Data Availability

GU2018_CL13 whole genome sequence and gene annotations can be obtained from the accession number AP029000 (https://www.ncbi.nlm.nih.gov/nuccore/AP029000).
