# Supplementary material for: TusDCB, a sulfur transferase complex involved in tRNA modification, contributes to UPEC pathogenicity
Source: Sci Rep. 2024 Apr 18;14:8978. doi: 10.1038/s41598-024-59614-2 (PMC11026471; doi:10.1038/s41598-024-59614-2)
Supplement: Supplementary file 1 — Supplementary Information. [file 41598_2024_59614_MOESM1_ESM.doc]

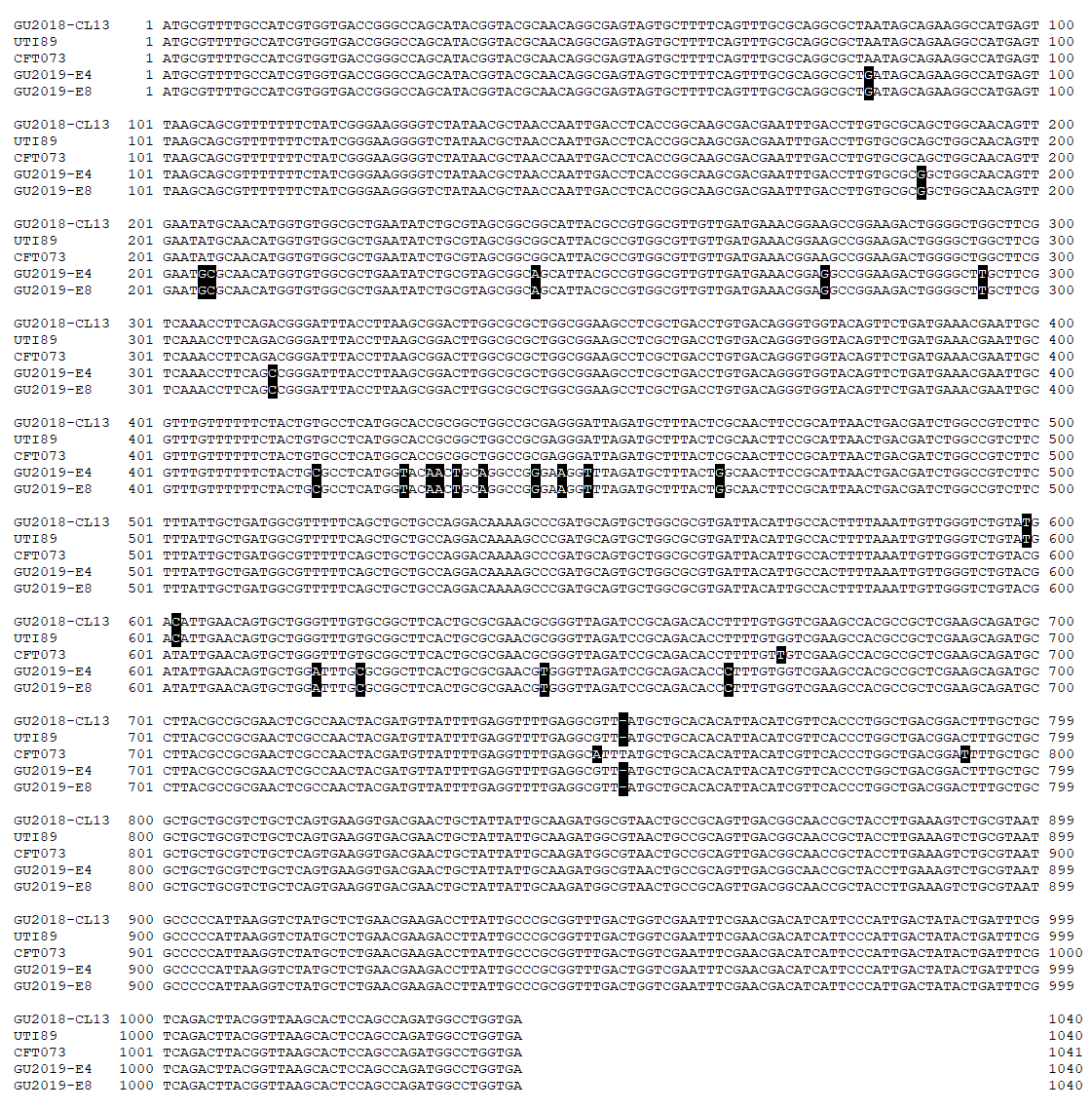


**Figure S1:** The nucleotide sequences of five UPEC strains of *tusDCB* genes. Their sequences were extracted and aligned them using ClustalW2 (https://doi.org/10.1093/bioinformatics/btm404). The accession numbers are as follows: UTI89 (CP000243), CFT073 (CP051263), GU2018_CL13 (AP029000). For GU2019-E4 and GU2019-E8, the sequences were determined using Sanger sequencing.
